# Differentiation of African Swine Fever Virus Strains Isolated in Estonia by Multiple Genetic Markers

**DOI:** 10.3390/pathogens12050720

**Published:** 2023-05-16

**Authors:** Annika Vilem, Imbi Nurmoja, Lea Tummeleht, Arvo Viltrop

**Affiliations:** 1 Institute of Veterinary Medicine and Animal Sciences, Estonian University of Life Sciences, 51006 Tartu, Estonia; 2 The National Centre for Laboratory Research and Risk Assessment, LABRIS, 51006 Tartu, Estonia

**Keywords:** African swine fever, molecular characterization, sequencing

## Abstract

The African swine fever virus (ASFV) was first detected in Estonia, in September 2014. In the subsequent three years, the virus spread explosively all over the country. Only one county, the island of Hiiumaa, remained free of the disease. Due to the drastic decrease in the wild boar population in the period of 2015–2018, the number of ASFV-positive cases among wild boar decreased substantially. From the beginning of 2019 to the autumn of 2020, no ASFV-positive wild boar or domestic pigs were detected in Estonia. A new occurrence of ASFV was detected in August 2020, and by the end of 2022, ASFV had been confirmed in seven counties in Estonia. Investigations into proven molecular markers, such as IGR I73R/I329L, MGF505-5R, K145R, O174L, and B602L, were performed with the aim of clarifying whether these cases of ASFV were new entries or remnants of previous epidemics. The sequences from the period of 2014–2022 were compared to the Georgia 2007/1 reference sequence and the variant strains present in Europe. The results indicated that not all the molecular markers of the virus successfully used in other geographical regions were suitable for tracing the spread of ASFV in Estonia. Only the B602L-gene analysis enabled us to place the ASFV isolates spreading in 2020–2022 into two epidemiologically different clusters.

## 1. Introduction

African swine fever (ASF) is currently one of the biggest threats to the global swine industry, as it causes serious economic losses due to the ban on the international trade of live animals and meat products from affected regions. It is caused by the ASF virus (ASFV), which is a large-enveloped double-stranded DNA virus and a member of the family Asfarviridae [1,2]. The ASFV originated in Africa and is still present in many African countries [3,4]. Starting from 2007, after the ASFV genotype II was introduced into the Caucasus region of Europe, the virus spread quickly west- and northwards, affecting domestic pigs and wild boar in eastern and central Europe and reaching the European Union (EU) in 2014, spreading to many of its member states [4,5].

Furthermore, ASFV has also spread to Asia, Oceania, and the Americas [4,6]. The absence of a vaccine and treatment, the global spread, and the recurring long-distance jumps of the virus within and between countries and continents make the control and eradication of ASF the biggest challenge for the global pig industry.

The ASF has spread among wild boar in Estonia since 2014 [7,8,9,10]. In the period from 2015 to 2017, 27 domestic pig outbreaks were also confirmed [7,11]. From 2018, the number of ASFV cases among wild boar started to decrease due to the drastic decrease in the wild boar population in the country. From February 2019 to the end of August 2020, there was an 18-month period in which no ASFV-PCR-positive wild boar or domestic pigs were detected, despite ongoing surveillance [12]. Since August 2020, ASFV-PCR-positive wild boar have re-emerged in three separate geographical regions in Estonia [12].

Similar to other EU countries, the genotyping of Estonian ASFV isolates on the basis of the partial sequencing of the B646L gene encoding the major ASFV capsid VP72 protein has grouped Estonian isolates within genotype II (GII), with 100% nucleotide identity in comparison with the Georgia 2007 reference strain [13]. However, in previous studies, several genetically distinct variants of the ASFV were detected in Estonia based on other genome regions. Based on the sequencing of the central variable region (CVR) of the B602L gene, a new ASFV genotype II CVR variant 2 (GII-CVR2) was detected in 2015. In addition, a single-nucleotide polymorphism (SNP) in the ASFV genotype II CVR variant 1 (GII-CVR1/SNP1) was found in 2017 [7]. Furthermore, in the north-east of Estonia, a unique subtype of ASFV GII was detected in 2014, characterized by a 15-kb deletion at the 5′ end of the genome [9,14].

Recently, the ASFV isolates from Western Poland were clustered based on sequences of the O174L, MGF505-5R, IGR I73R/I329L, and K145R genome regions, enabling the definition of the geographical origin of the virus isolates [15]. These gene regions have also been useful for the molecular typing of ASFV isolates in Germany since the introduction of ASFV GII in 2020. The virus isolates were clearly distinguished from those spreading in other affected areas in Europe and the Caucasus region, enabling the confirmation of the virus’ origination in Western Poland [16,17]. Furthermore, a recent study conducted by the European Union Reference Laboratory for ASF revealed the O174L variant 2 in Romania and the K145R variant 2 in Lithuania, Ukraine, and Romania [5].

Based on these findings, the molecular typing of Estonian ASFV isolates using the aforementioned molecular markers was undertaken with the aim of describing the genetic variability of our ASFV isolates and, possibly, explaining the origins of the newly re-emerged virus in Estonia. The B602L gene was also added to the analysis as the most variable locus in previous studies [7]. To answer the question of whether these new findings were due to new introductions of ASFV or whether they were the remnants of the previous epidemic wave, the molecular typing of the virus isolates involved is necessary.

## 2. Materials and Methods

### 2.1. Sampling

The sampling of hunted wild boar and domestic pigs in Estonia was in accordance with EU legislation Council Directive 2002/60/EC and the Estonian ASF control program, and it was executed by the competent authority (the Agriculture and Food Board). The sampling scheme for ASF surveillance in wild boar has remained unchanged from the beginning of the epidemic and was described in more detail previously [7,8,9,11]. Briefly, blood samples were collected by hunters from all hunted wild boar for ASFV and antibody detection. From wild boar found dead, samples from organs, such as the spleen, kidneys, lymph nodes, or tubular bone were collected by the official veterinarian for ASFV analysis. Sampling in domestic pigs was focused on the testing of organ samples collected regularly from dead and sick animals (passive surveillance).

### 2.2. Selection of Isolates

In total, 146 ASFV polymerase chain reaction (PCR)-positive samples were selected for further studies (Table 1, Supplementary Table S1). The aim of selection of the samples from wild boar was to cover all affected areas over the period of 2015–2022. Of these samples, 104 from 14 affected counties from the period of 2015–2018 (the “epidemic” phase of the ASF spread) were selected. The remaining 43 samples (42 from wild boar and 1 from a domestic pig from an outbreak farm) originated from the period after the re-emergence of ASFV in the wild boar population, from August 2020 to the end of 2022, including isolates from all seven newly affected counties (Table 1). Detailed information on the ASFV GII isolates selected to study genetic variability in Estonia are presented in Table S1.

### 2.3. Genome Detection and Amplification of Gene Regions by Conventional PCR and Sequence Analysis

The ASF genome detection was performed as described previously by Vilem et al. [7]. The regions O174L, MGF505-5R, IGR I73R/I329L, K145R, and CVR of the gene B602L were used for the further characterization of the virus strains. The amplification of the O174L, MGF505-5R, IGR I73R/I329L, and K145R was performed using the primers described by Mazur-Panasiuk et al. [15]. A PCR was performed with the 5× HOT FIREPol^®^ Blend Master Mix with 15.0 mM MgCl_2_, according to the manufacturer’s instructions. Each gene region was amplified in a separate PCR run. Briefly, 10.4 µL of nuclease-free water, 4 µL of 5× HOT FIREPol^®^ Blend Master Mix, and 0.8 µL of each of the forward and reverse primers targeting the gene regions of interest with a final concentration of 0.4 µM were pooled together as the master mix. Finally, 4 µL of aliquot of extracted DNA from the sample was added to 16 µL of the PCR master mix. Cycling conditions were one cycle at 95 °C for 15 min, followed by 40 cycles consisting of denaturation for 30 s at 95 °C, annealing for 30 sec at temperatures of 53 °C, 52 °C, 50 °C, and 50 °C for the genes MGF505-5R, O174L, IGR I73R/I329L and K145R, respectively, and extension for 1 min at 72 °C. A final extension for 10 min at 72 °C was added at the end of the PCR.

The amplification of the B602L gene was performed for isolates collected from August 2020 to the end of the study period, as described previously by Vilem et al. For isolates from the period of 2014 to 2018, sequence information regarding the B602L gene was available from a previous study [7]. The PCR products were visualized on 1.5% agarose gel. Further sequencing of the DNA samples collected from electrophoresis gel was performed by a service provider (University of Tartu, Estonia, Institute of Genomics) with the Applied Biosystems^®^ 3130xl Genetic Analyzer, using a two-directional procedure. Nucleotide sequences were edited and analyzed using MEGA7.0.26 software with the ClustalW alignment [18]. The K145R, O174L, and MGF505-5R sequence analyses were performed in comparison with the Georgia 2007/1 reference sequence (GenBank accession number FR682468.2) [13]. For the B602L gene-sequence alignment, the representatives of the previously detected Estonian CVR variant strains (GenBank accession numbers MT647548 and MT647527) [7], Lithuanian LT14/1490 (GenBank accession number KJ627204) [2], and Russian CVR variant strains (GenBank accession numbers ON098036, ON098025, ON098023 and ON098019) [19] were added. For IGR I73R/I329L analysis, the index case of ASFV in the EU was included (GenBank accession MK628478) [20] to sequence alignment.

The nucleotide sequences of the Estonian ASFVs belonging to GII-CVR1/SNP4 variants were deposited in GenBank (Accession numbers from OQ675547 to OQ675557, Table S1). In addition, all sequences generated in this study belonging to the same Georgia-type variant or GII-IGR I73R/I329L variant 2 are available upon request from the National Centre for Laboratory Research and Risk Assessment, LABRIS https://labris.agri.ee/en (accessed on 31 March 2023).

### 2.4. Mapping Software

A map with the virus-isolate locations was created using Stata/BE 17.0 (StataCorp LP, College Station, TX, USA) command grmap (visualization of spatial data). A map of Estonia, the “Administrative and Settlement Division” Counties map, was obtained from the Estonian Land Board website [21]. The map’s coordinate-reference system (CRS) was set to EPSG:3301—the Estonian Coordinate System of 1997. European map was downloaded from Peter Hermes Furian iStock, Getty Images [22].

## 3. Results

### 3.1. Sequencing of the IGR I73R/I329L, MGF505-5R, K145R, and O174L Gene Regions

The number of selected and successfully sequenced isolates per county and by gene region are presented in Table 1. The sequences of the IGR I73R/I329L were obtained from 130 selected isolates. The sequencing failed in 16 of the isolates. All the successfully sequenced isolates belonged to IGR I73R/I329L variant 2, which was the predominant variant strain in EU countries [2,7,15]. The sequencing of the MGF505-5R was successful for 123 isolates and failed in 23 cases. All the sequenced isolates were 100% similar to each other and homologous with the Georgia 2007/1 reference strain. The sequencing of the K145R-gene region was successful from one hundred and thirty-seven samples and failed in nine. The sequence alignment of the K145R-gene region of the Estonian isolates revealed a 100% homology both between each of the isolates and with the Georgia 2007/1 reference strain. The sequencing of the O174L failed in 31 samples, and results were obtained for 115 isolates. The sequence alignment of the O174L gene region showed a 100% homology both between each of the isolates and with the Georgia 2007/1 reference strain.

### 3.2. Sequencing of the B602L Gene

**Figure 1:**
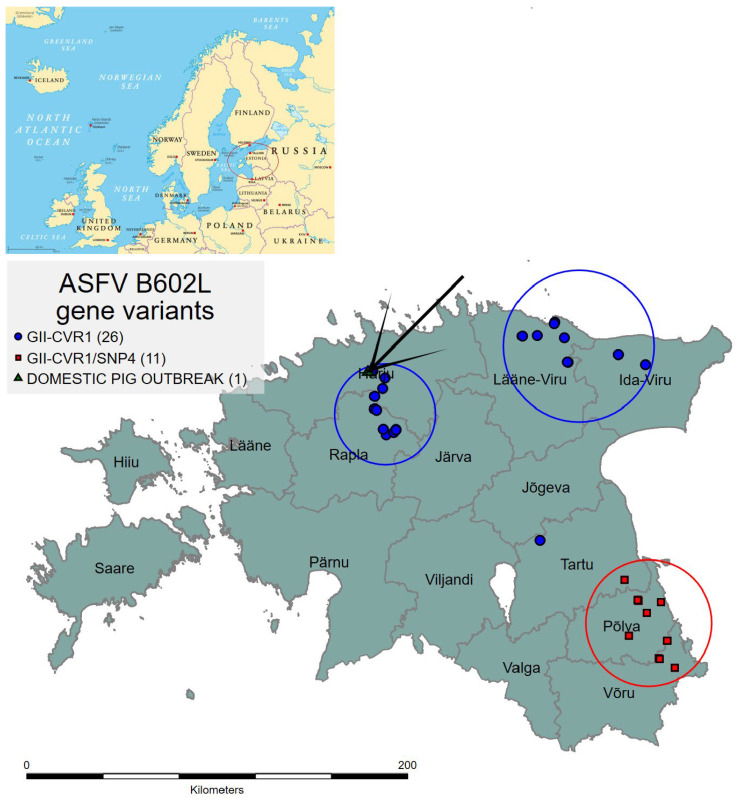
Illustration of two epidemiological clusters based on the ASFV B602L-gene-variant strains detected in Estonia in 2020–2022. ASFV isolates belonging to the NNE and SE clusters are marked in blue and red, respectively. An outbreak in domestic pigs is marked in green and indicated by an arrow. The number in parentheses indicates how many strains the corresponding variant was detected on.

The sequencing of the B602L gene of the isolates from the period of 2020 to 2022 was successful in thirty-eight isolates and failed in four (Table 1). The sequence alignment confirmed that the Estonian isolates from this period were clustered into two geographically defined groups: the north and north-eastern cluster (NNE, including Rapla, Harju, Lääne-Viru, and Ida-Viru counties) and the south-eastern cluster (SE, including Võru, Põlva and Tartu counties) (Figure 1).

All the isolates from the NNE cluster (*n* = 27, including wild boar and domestic pigs) belonged to the GII-CVR1 and were homologous with the Georgia 2007 reference sequence (Georgia-type variant).

**Figure 2:**
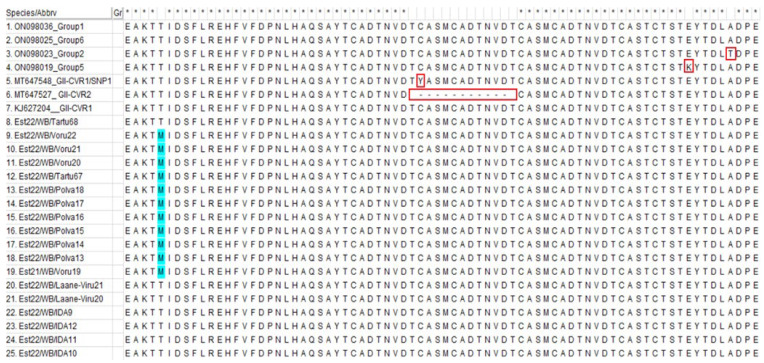
Amino-acid alignment of part of the GII-CVR-variant strains used in study. The amino-acid change in the strains from the south-east of Estonia during the period of 2021–2022 is marked in blue. Other variants detected previously within the B602L gene are marked in red. * indicates the lack of differences between the aligned isolates.

The sequence alignment of the isolates from the SE cluster (*n* = 11) revealed a single-nucleotide polymorphism (SNP) at position 412 in a complete B602L gene. Thus, a new GII-CVR1/SNP4 was confirmed. The nucleotide change resulted in an amino-acid change, whereby cytosine (C) was replaced with tyrosine (T) (Supplementary Table S2). The aforementioned nucleotide change was a non-synonymous SNP, which also resulted in an amino-acid change (M instead of T) (Figure 2).

## 4. Discussion

The molecular characterization of pathogens has become an indispensable part of epidemiological investigation of any infectious animal disease, including ASF. Partial Sanger sequencing is widely used in affected regions for the screening of circulating ASFV strains. Although the ASFV genome is considered to possess a relatively low mutation rate [23,24], previous studies have revealed several intragenotype variations in Estonia [7,14]. In 2014, the attenuated phenotype of the ASFV was detected in the north-east of Estonia [14]. This finding supported the hypothesis that two entries of the virus into Estonia occurred, separated by time and region, based on observations and the analysis of the case data from the field [8]. A subset of ASFV isolates from the period of 2014–2019 from various parts of the country was screened for a 15-kb deletion in LABRIS to determine the spread of the attenuated phenotype, but no mutant variant was identified in subsequent years. This suggests that the ASFV with the 15-kb deletion is most likely extinct. Furthermore, two unique variant strains within B602L and their limited spread in time and space, were confirmed previously. One of the detected variants, GII-CVR1/SNP1, was also responsible for one domestic pig outbreak in 2017. Spatial investigations revealed the circulation of GII-CVR1/SNP1 among wild boar only a month before the domestic pig outbreak was confirmed in the area [7]. This finding supported the opinion that the infection of wild boar is the main risk factor in the infection of domestic pigs [11].

In the current study, the aim was to investigate whether additional genetic variability could be found in Estonian ASFV isolates in genome loci recently shown to be useful molecular markers for strain differentiation in Poland, Germany, and the Russian Federation (Kaliningrad region) [15,16,25,26] and, if possible, to identify the origin of the ASFV that has re-emerged since August 2020 in Estonia.

Some of the ASFV isolates could not be sequenced, mainly due to the low viral load (see ct values in Table S1). Furthermore, the DNA extracted from the samples after they were collected and stored for further studies was used for sequencing. Therefore, in some cases, DNA freezing and storage may have affected the quality of the amplification and sequencing, despite the viral load, because some of the samples used were two to seven years old. The highest number of failures occurred in the sequencing of O174L. Non-specific amplification was observed, which was not seen during the amplification of the other investigated regions. This may have been an additional factor affecting the quality of the O174L sequencing, as the amplification product was not extracted from the gel for sequencing. Nevertheless, the exact cause of the sequencing failures remains unclear.

The results indicated that the O174L, MGF505-5R, and K145R sequencing did not reveal differences between the Estonian strains and the Georgia 2007/1. Furthermore, the sequencing of IGR I73R/I329L confirmed that the Estonian isolates share the 10-nucleotide insertion and belong to IGR variant 2, which is the most common variant in the EU [2,5]. In a recent study conducted by Mazloum et al., the sequencing of the complete K145R revealed two simultaneous SNPs, one unique to the Kaliningrad region in comparison with Georgia-type and the second unique to the SNP and connected to the K145R variant 2 [27]. The primers used to sequence the K145R in this study did not cover the SNP described in Kaliningrad.

These results indicate that not all the molecular markers successfully used in some regions are equally useful in others. This indicates that the mutation rates may vary substantially between the isolates, and that the genome regions that exhibit increased mutation rates vary in different subpopulations of the virus. This may mean that the genome loci and the genes suitable for the molecular differentiation of ASFV isolates are specific to certain geographical regions sharing the subpopulation(s) of the virus.

Previously, most of the variability between the isolates in Estonia was found in the B602L genome region [7], and this was also the case for the more recent isolates, indicating that this might be a locus to which greater attention may be directed in the future. The results of the B602L sequencing in this study revealed no circulation of either the GII-CVR2 or the GII-CVR1/SNP1 variant strain, which was in line with the conclusions drawn by Vilem et al., who found that the aforementioned variants became extinct after a period of limited circulation. Although the extinction of the GII-CVR2 variant could be explained by its somewhat reduced virulence [28], the reason for the disappearance of the GII-CVR1/SNP1 variant remains unclear. Regardless of whether it was a random event, the occurrence of a reverse mutation, or an amino-acid change, the virus became less successful in infecting hosts. The questions relating to the cause of this change are as yet unanswered.

The GII-CVR1/SNP4 variant detected for the first time in the framework of this study needs further investigation, since the relevance of this substitution to the changing phenotypic properties of the virus remains unknown. Nevertheless, this change places the ASFV isolates from the south-east of Estonia in a separate cluster, and they are therefore probably not connected to the virus circulating in the north and north-east of Estonia. Temporal and spatial investigations into ASFV circulation after a one-and-a-half-year break led to a first finding, in August 2020, in Rapla county (NNE cluster). This location is geographically far from the borders, which suggested, based on the molecular investigations, which showed a 100% homology with most previous Estonian isolates, that the ASFV circulating in the NNE areas was likely to be a remnant of the previous epidemic of the virus. The re-emergence of ASFV-positive wild boar after an 18-month period in which there were no such findings, despite ongoing surveillance, remains a mystery. The possible reasons were analyzed and discussed thoroughly by Schulz et al. [12]. In brief, the re-emergence may indicate that the virus is capable of persisting in populations with very low density (~0.5 WB per km^2^), with a very low prevalence (<1%), for a relatively long period (>1 year). However, there is no good evidence to support the biological mechanisms underlying this persistence. The second option is that the virus was re-introduced by humans, and that the virus originated from old, frozen wild-boar meat, leftovers of which were discarded in a forest. The beginning of the decrease in wild-boar density in 2019 and 2020 provided favorable conditions for the virus to spread again.

On the other hand, the ASFV findings from the SE of Estonia, starting from autumn 2021, suggest that they are likely to represent a new entry of the virus from the neighboring Russian Federation (RF). This can be suspected because of the fact that previous findings of ASFV in the area originated from February 2018, and the first re-emerging cases in the SE of Estonia, in autumn 2021, were detected less than 10 km from the border with the RF. The fact that all the isolates from this area share the same mutation within the B602L gene and belong to the same GII-CVR1/SNP4 variant, which has not been discovered anywhere else in Estonia, support this hypothesis. However, no information regarding the B602L-gene sequences from the RF from the period of 2018–2022 is available to confirm this. The investigation into the ASFV B602L gene sequences in the RF was performed during the period of 2013–2017, but according to the sequence alignment performed in the current study, the GII-CVR1/SNP4 variant was not present among these strains (Figure 2, Table S2) [19,27]. Therefore, whole-genome-sequence data are needed to further clarify the origin of the virus.

Other variant strains, GII-CVR1/SNP2 and GII-CVR1/SNP3, were previously detected in Poland and Lithuania, respectively, and also have point mutations in other positions [5].

The results in this study demonstrate the slow expansion of the GII-CVR1/SNP4-variant strain in the SE counties of Estonia, spreading towards the west and north-west, covering an area with a diameter of approximately 65 km by the end of 2022.

In December 2022, the second ASFV-positive wild boar after the re-emergence was detected in Tartu County, ~50 km away from the first finding. However, the genotype of the detected virus in Tartu County in December 2022 was the original and belonged to the same cluster as the isolates from the NNE. This case was more than 100 km away from the nearest ASFV findings in the NNE cluster and most likely represents a human-mediated long-distance jump of the virus. However, it could be another example of the silent circulation or persistence of the virus in a wild-boar population or environment over a long time period (>one year) without evidence from tested wild boar.

## 5. Conclusions

In the current study, we demonstrated that not all the molecular markers, such as O174L, MGF505-5R, K145R, and IGR I73R/I329L, successfully used in other geographical regions (e.g., Poland and Germany) are useful in other territories. It is apparent that there are subpopulations of the virus for which the mutation rate in these genome regions is higher, making it possible to track the virus in time and space, but this may not be possible in other geographical areas, such as Estonia, as suggested in the current study. One of the proven markers for clustering Estonian ASFV isolates is the B602L gene. As a result of this study, a new GII-CVR1/SNP4 variant was confirmed, and Estonian ASFV isolates from the period of 2020–2022 were divided into two clusters. However, new molecular markers suitable for the discrimination of Estonian ASFV are necessary. The whole-genome sequencing (WGS) of the virus allows the identification of new genetic markers and the tracing of the spread of the virus. A follow-up study, using a targeted WGS analysis, is ongoing.

## Figures and Tables

**Table 1:** Number of ASFV isolates selected from the period 2015–2022 and successfully sequenced per county by genome region.

County		No. of Isolates Selected	No. of Successfully Sequenced Isolates by Genome Region					No. of Isolates Selected for B602L *
			IGR I73-I329L	MGF505-5R	K145R	O174L	CVR B602L	
Harju **		18	16	14	17	14	5	5
Ida-Viru		9	6	6	9	5	4	4
Jõgeva		5	5	5	5	5		
Järva		4	3	3	4	2		
Lääne		7	6	6	6	5		
Lääne-Viru		19	18	13	15	14	7	9
Põlva		10	10	10	10	10	6	6
Pärnu		8	7	7	7	6		
Rapla		21	18	17	20	17	10	11
Saare		9	9	8	9	7		
Tartu		11	8	11	11	9	2	2
Valga		6	6	6	6	6		
Viljandi		7	7	6	7	6		
Võru		12	11	11	11	9	4	5
Total	*n*	146	130	123	137	115	38	42
	%	X	89.0	84.2	93.8	78.8	26.0	X

* In total, 42 isolates were selected from the period of August 2020 until the end of 2022. ** One isolate originated from the domestic pig from the outbreak farm.

## Data Availability

Data is contained within the article and Supplementary Materials. New data generated in this study are available in GenBank https://www.ncbi.nlm.nih.gov/genbank (URL Accessed on 13 May 2023) (Accession numbers from OQ675547 to OQ675557).

## Supplementary Materials

The following supporting information can be downloaded at: https://www.mdpi.com/article/10.3390/pathogens12050720/s1. Supplementary Table S1: African swine-fever virus genotype II isolates selected to study genetic variability in Estonia. Supplementary Table S2: Nucleotide-sequence alignment of the partial B602L gene representing the differences in positions 411–629 of the complete gene.
